# Healthcare Practitioners' Perceptions of Quality‐of‐Life Tools in Head and Neck Surgical Oncology: A Qualitative Descriptive Content Analysis

**DOI:** 10.1002/lio2.70531

**Published:** 2026-08-20

**Authors:** Keshav Kumar Gupta, Jameel Muzaffar

**Affiliations:** ^1^ Department of ENT Surgery University Hospitals Birmingham NHS Foundation Trust. Queen Elizabeth Hospital Birmingham UK; ^2^ Honorary Senior Research Fellow. Department of Applied Health Sciences University of Birmingham Birmingham UK

**Keywords:** content analysis, head and neck, head and neck cancer, qualitative research, quality of life

## Abstract

**Objectives:**

Quality of life (QoL) outcomes are becoming increasingly important in the era of patient‐centered medicine. This is especially true in head and neck surgical oncology due to the life‐altering effects of major head and neck surgery. This study aimed to gain in‐depth feedback surrounding the awareness, perceptions, and utilization of QoL tools in head and neck surgical oncology among the multidisciplinary team (MDT).

**Methods:**

A qualitative descriptive content analysis of semi‐structured interviews from 10 members of the head and neck surgical oncology MDT.

**Results:**

The overarching categories from the interviews were of healthcare practitioners (HCPs) expressing that they thought the assessment of QoL to be important in this patient group and that this was important for clinical practice, patients, and in research. While most respondents assessed QoL, this was largely done on an informal basis, with very few regularly employing formal QoL tools. Various barriers to their use in terms of patient, clinician, and logistical factors were explored.

**Conclusions:**

Overall, this study adds to the literature by providing an important analysis of perceptions of QoL and formal tools in head and neck surgical oncology from the underexplored perspective of HCPs in this MDT. While clinicians across the MDT generally viewed QoL assessment positively, implementation of structured tools remained inconsistent and was influenced by structural barriers. These findings suggest that future efforts should focus on developing feasible, clinically embedded approaches to QoL integration within this context.

**Level of Evidence:**

N/A.

## 1 Introduction

Quality of life (QoL) outcomes are becoming increasingly important in the era of patient‐centered medicine in order to tailor treatment options and management strategies for patients. In head and neck surgical oncology, such outcomes can hold even more significance due to the life altering effects of major head and neck surgery [1, 2]. While radical surgery can improve survival, this must be balanced with the subsequent, and often profound, effect on patients' QoL. This impact can be across multiple avenues including physical (e.g., pain, xerostomia, mucositis), functional (e.g., dysphagia, dysphonia) and psychosocial (e.g., depression, disfigurement, isolation) outcomes [3]. Assessment of QoL in head and neck cancer patients is therefore critical in terms of evaluating treatment options and response to treatment, as well as planning and monitoring rehabilitation.

In head and neck surgical oncology, there are a multitude of general and specific validated QoL instruments that can be utilized [4]. The management of head and neck cancer patients is centered around a multidisciplinary (MDT) approach due to the complex interactions of multiple systems that need to work together to optimize patient function post‐treatment. Key members include surgeons (usually otolaryngology (ENT) and oral and maxillofacial (OMFS)), clinical nurse specialists (CNS), speech and language therapists (SLT), and dietitians. While there have been some studies surrounding patient perceptions of their QoL [5, 6, 7], to date, there is little research exploring health care practitioner (HCP) perceptions of validated QoL tools with respect to their use and usefulness from an interview‐based and multi‐disciplinary standpoint. Given the fact that the MDT is central in managing head and neck cancer patients, there is scope for many different HCPs to utilize various QoL tools related to their domain. This study is therefore important as it aims to fill a gap in the current literature and can help demonstrate how QoL tools are being used or not used in a healthcare context where QoL is very important to patients.

The aim of this study is to therefore gain in‐depth feedback surrounding the awareness, perceptions, and utilization of QoL tools in head and neck surgical oncology among the MDT. For the purposes of this study, the impact of other non‐surgical treatment options, such as radiotherapy, was not explored.

## 2 Materials and Methods

### 2.1 Study Design and Setting

The study was conducted at a UK tertiary referral and teaching hospital in the head and neck surgical oncology department. This encompasses a large and varied case mix including various HCPs (ENT and OMFS surgeons, CNS, SLT, dietitians, and altered airway nurses (AAN)). These HCPs are involved in direct patient‐facing care either on the ward, in clinic, theater, or as any combination of the three. Each professional group contributes in different ways toward patient care and works closely together in various settings. There is a weekly MDT discussion of cancer patients to review and collaborate on treatment and management plans.

A qualitative description‐based content analysis was planned through semi‐structured interviews of HCPs in the head and neck surgical oncology MDT. The study was informed by a constructivist orientation, recognizing that HCPs may hold multiple subjective understandings of QoL and its clinical relevance. However, the analysis aimed to remain close to participants' accounts and prioritized descriptive over highly interpretive analyses. The study was reported according to the Standards for Reporting Qualitative Research (SRQR) checklist [8].

### 2.2 Ethical Considerations

The study Sponsor was University Hospitals Birmingham NHS Foundation Trust and was prospectively registered with Integrated Research Application System (IRAS) (343373) who confirmed its exemption from Research Ethics Committee (REC) approval due to no patient involvement. Written informed consent was obtained from each participant prior to interview. No compensation, payment or reimbursement of any kind was offered or given to any participant. Transcripts were anonymized by assigning identification numbers (e.g., participant 1) for each participant. All electronic data were stored on Sponsor‐approved password‐protected NHS devices and managed using NVivo 14 (QSR International, Melbourne, Australia) data management software.

### 2.3 Sampling

Purposeful sampling was used to obtain interviews from a wide and representative range of HCPs from the head and neck surgical oncology MDT. Eligibility criteria included: (1) patient facing involvement (clinic, ward, theater or mixed) with head and neck surgical oncology patients, (2) ability to participate in an interview conducted in English. Exclusion criteria were: (1) clinicians involved exclusively in thyroid oncology, (2) study authors. No exclusion criteria beyond these requirements were applied. At our center, this would encompass all members of the head and neck surgical oncology wider MDT (excluding the study authors) ENT and OMFS surgeons, SLT, AANs, CNSs, and dietitians. Sampling aimed for around 8–12 participants and was guided by information power, role diversity, and anticipated data richness, rather than a predefined requirement for thematic saturation. This was deemed appropriate based on previous similar studies [9, 10, 11, 12] and was flexible, with further interviews planned dependent on the quality of previous interviews and if further areas relevant to the study aims required exploration.

Recruitment was prospective beginning on 20th January 2025 and ending on 21st February 2025. Participants were approached either via email or verbal discussion face to face by either author, and invited to respond within 7 days. No posters or other paraphernalia were used to aid recruitment. A total of 10 HCPs were approached, all of whom agreed to participate in the study. All participants received a standardized and pre‐designed Participant Information Sheet (PIS) prior to providing signed written consent before conducting their interview. Following the initial 10 interviews, both authors agreed, through discussion, that the dataset provided sufficient informational depth and breadth to address the study aims; therefore, no further recruitment was pursued. In addition to interviews, data was collected on participant demographics including their role and years of experience within their current role.

### 2.4 Interview Design

All interviews were conducted on a 1:1 basis over Microsoft Teams (Microsoft, Redmond, USA) using secure hospital e‐mail address logins with a single interviewer (K.K.G.) and the participant. The Microsoft Teams recording function was used to auto‐transcribe each interview that was manually checked for accuracy. All participants provided written informed consent prior to taking part in their interview in the presence of the lead author (K.K.G.) that was documented on a standardized and pre‐designed consent form. Participants were made aware that they were able to terminate the interview at any point or redact any statements made. This would result in the interview not being used in the final analysis. Interviews followed a semi‐structured format guided by a prior interview protocol (Supporting Information A). This was organized around four broad topics relevant to the study aims, namely; (1) understanding and awareness, (2) importance, (3) utilization, (4) usefulness of QoL and QoL tools. This served to structure the interviews rather than predefine any analytic categories and participants were free to raise topics not anticipated by the protocol. No participant was able to review this protocol prior to their interview to minimize pre‐thought or rehearsed responses.

The interview protocol was developed iteratively by both authors based on the study aims and existing literature surrounding QoL assessment and PROM utilization in this field. The protocol enabled flexibility in terms of follow‐up questions and probes in order to collect open‐ended responses to fully explore participants thoughts and feelings surrounding QoL tools in this context. Transcript review by participants (member checking) was not undertaken, as the study prioritized close descriptive analysis of interview content and aimed to minimize participant burden.

Both authors work in the same institution as the participants. Therefore, to mitigate potential hierarchical influences, participants were informed (verbally and through the PIS) that there were no right or wrong answers and participation was wholly voluntary and could be withdrawn at any time without reason. They were also informed that the answers given would not be used to judge or assess their practice. Furthermore, the interview was conducted by the more junior author who aimed to further minimize hierarchical influence through the use of open‐ended questioning, reflexive journaling, and avoidance of leading prompts.

### 2.5 Data Analysis

A qualitative content descriptive approach was employed to enable a close‐to‐data and low‐inference account of HCP perceptions regarding QoL tools when assessing head and neck surgical oncology patients [13, 14, 15]. The content was analyzed using a conventional qualitative content method as described by Hsieh & Shannon [16], selected as it best supports inductive category development directly from interviews to remain close to the data with low levels of abstraction, aligning with our study aims.

Following transcription of all interviews, a single author (K.K.G.) read all transcripts at least twice to achieve familiarization with the data. An initial round of open coding was conducted by assigning labels to segments of text that were meaningful and relevant to the research aims. Codes were generated from participant's accounts and grouped into sub‐categories generated inductively. Transcripts were then re‐read to allow annotations and memos with reflections and then grouped into descriptive sub‐categories to allow further exploration of categories within them. These were compared and collapsed to form higher‐order categories representing shared perceptions and themes across participants. Peer‐debriefing was undertaken to enhance analytic credibility with the senior author (J.M.) to review the coding and categories with any non‐conforming cases or areas of debate through discussion to ensure interpretations remained grounded in the full data set.

### 2.6 Reflexivity and Trustworthiness

Reflexive journaling was undertaken at various points in the data collection period to document and bracket any pre‐conceived assumptions held by the authors. The interviewer (K.K.G.) is an ENT senior resident working in the same institution as the senior author (ENT consultant) and the participants. Both authors acknowledged that their preconceived and overall, largely favorable, views regarding QoL tools in clinical practice had the potential to influence analysis, as well as their prior clinical interactions with all participants to a varying degree. The peer debrief process between both authors helped to challenge interpretations and enhance analytical reflexivity. Measures taken to minimize hierarchical influence during interviews are detailed earlier.

Several strategies were employed to enhance trustworthiness and rigor. Regular peer debrief (including review of coding framework, categories and interpretations) allowed interrogation and critique of the initial analysis to help identify alternative interpretations and improve overall credibility. In addition, rich and representative excerpts were included in the manuscript to capture the breadth of perspectives and provide evidence of the emergent categories. Furthermore, any negative case analysis was included and highlighted to strengthen the credibility of the final analysis. We provide a thick description of the study setting and MDT workflow to help with study transferability.

## 3 Results

A total of 10 interviews were conducted with 10 different HCPs from the head and neck surgical oncology MDT at our single center between January and February 2025 (Table 1). No participant withdrew from the study. The overall categories that emerged from the interviews could broadly be categorized into understanding and awareness, importance, usefulness, utilization and barriers of QoL and QoL tools. The average interview length was 11 min 48 s (range 7 min 6 s—14 min 42 s).

**TABLE 1:** Participant characteristics.

Participant number	Role	Years of experience in current role (at time of interview)
1	ENT surgeon	> 15
2	ENT surgeon	> 15
3	ENT surgeon	< 5
4	OMF surgeon	10‐15
5	OMF surgeon	> 15
6	Altered airway nurse	< 5
7	Speech and language therapist	5‐10
8	Speech and language therapist	< 5
9	Clinical nurse specialist	< 5
10	Dietitian	< 5

Abbreviations: ENT, ear, nose and throat (otorhinolaryngology); OMF, oral maxillofacial.

### 3.1 Understanding and Awareness

Overall, most participants had a general understanding and awareness of what QoL means. Many participants commented on QoL meaning a patient's ability function as they would like. For example, responses included, “how well they are living their life” (Participant 4‐OMFS), “patient's ability to do the things that they want to do” (P3‐ENT), *“day‐to‐day function”* (P5‐OMFS), “being able to do the things that they want to do and being able to do them comfortably” (P6‐AAN), “the enjoyment that they can have out of being able to complete their activities of daily living” (P9‐CNS). In addition, there were many responses about QoL being a subjective perception that is individual to each patient. For example, “it's the patient's perception or how well they're living” (P4‐OMFS), “it's really a parameter that's assessed by the patient and their own perception of what it is” (P5‐OMFS), “it's really subjective” (P8‐SLT), “I suppose it's very individual to all patients and their carers, families, loved ones” (P9‐CNS). There were also comments about QoL being about how much patients can enjoy their life post treatment, “their enjoyment in life” (P7‐SLT), “the ability to function post treatment” (P10‐D) and that this can encompass many different factors, such as physical and mental well‐being (P1‐ENT), socializing, eating and drinking and going on holidays (P8‐SLT).

### 3.2 Importance

Most participants commented on their feelings surrounding assessment and consideration of QoL to be important. This was in the context of planning and offering treatment options and allowing patients to make informed decisions. For example, “yeah, definitely because they need to be able to make informed decisions about their care and if there's an aspect of it that's going to really negatively impact their life, they need to be able to take into account the impact of that treatment” (P8‐SLT), “yeah, it's very important. I think whatever we decide…it should eventually translate into quality of life and we need to balance the treatment versus quality of life” (P1‐ENT), *“it's really critical that you also take into consideration what their quality of life is likely to be afterward* (post treatment) *and the most important bit is to educate them* (patients) *and manage their expectations”* (P4‐OMFS).

When comparing the importance of consideration of QoL compared to participant's non‐oncological practice, the majority of participants felt that QoL was more important to consider in their oncology patients. For example, *“the impact of the treatment normally has a much higher impact on quality of life* (compared to non‐oncological treatment),*”* “the long lasting implications of any treatment are massive on quality of life so it's certainly something that I think about more in my oncology practice” (P3‐ENT), “I don't think there are many subspecialities within maxillofacial that would have as much of an impact on quality of life….you're carrying out radical surgery in people that will affect their facial appearance, that will affect their speech, their swallowing…things that people really consider very important to integrate back into society” (P5‐OMFS), “I do consider quality of life…I think with the cancer population its almost that little bit more important because…they may have limited time left” (P7‐SLT).

However, two participants commented on QoL being less important in their oncology practice as there is more of a focus on cure. For example, “you have a patient who's got cancer, so its life and death, it's not a quality for him, it's a life” (P1‐ENT), *“so I think it's* (quality of life) *probably less important in the ENT oncology because the emphasis is on the cure first”* (P2‐ENT). Notably, these views were expressed by the most experienced ENT surgeons in the sample, highlighting potential variation in how survival and QoL considerations are prioritized.

### 3.3 Usefulness

**FIGURE 1:**
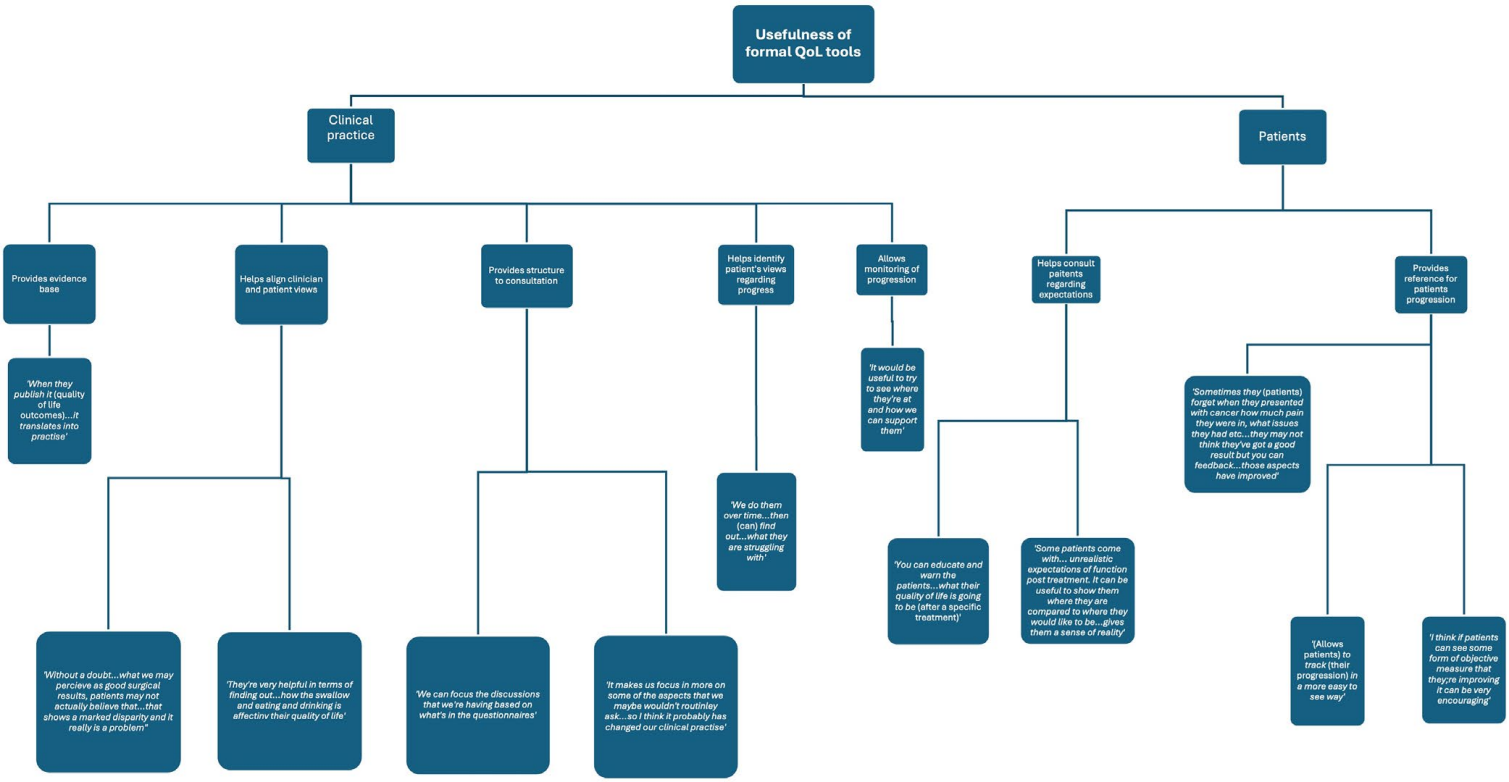
Reported usefulness of formal quality of life (QoL) tools.

When exploring participant thoughts regarding the usefulness of formal QoL tools, most expressed that they felt they were largely useful for their clinical practice and for patients. Figure 1 summarizes the general categories that emerged from this topic. Participants also commented on the fact that the use of formal QoL tools can be useful from a research standpoint, in order to provide an evidence base to guide clinical practice, gather data and to evaluate another important outcome measure other than survival. For example, “I think it is important we collect the data… for retrospective audit purposes” (P2‐ENT) “it gives you kind of quantitative data…that we can kind of track and look at…and hopefully see trends” (P6‐AAN), “*it* (collecting quality of life data) *helps with that evidence base”* (P7‐SLT). There were some responses that included strong language to convey their agreement that the use of QoL tools are important in research; *“yes certainly in research it'll be useful…it's often overlooked…there's definitely less attention paid on quality of life* (compared to survival outcomes) *and I think for research it'll be really helpful”* (P3‐ENT).

### 3.4 Utilization

During the interviews, only two participants commented on their regular use of formal QoL tools. The rest of the participants stated that they did not use formal tools. Despite this, all participants discussed how they evaluated patient QoL informally during their consultation. For example, “I definitely assess it informally every time patient's come back for a post treatment visit…I try and discuss all aspects of their quality of life” (P3‐ENT), *“informally I do* (assess quality of life), *I like to know everything about a patient…you are sort of assessing it all the time”* (P4‐OMFS), “informally…we'll have discussions with them about what they are doing day‐to‐day, how they're managing” (P6‐AAN).

The two participants who used formal QoL tools regularly were both SLTs (participants 7 and 8). They both also assessed patient QoL informally. The main formal tool used routinely, and at different time points, was the MD Anderson Dysphagia Inventory (MDADI), used to evaluate swallowing function using 20 questions across four domains (global, emotional, functional, and physical).

### 3.5 Barriers

**FIGURE 2:**
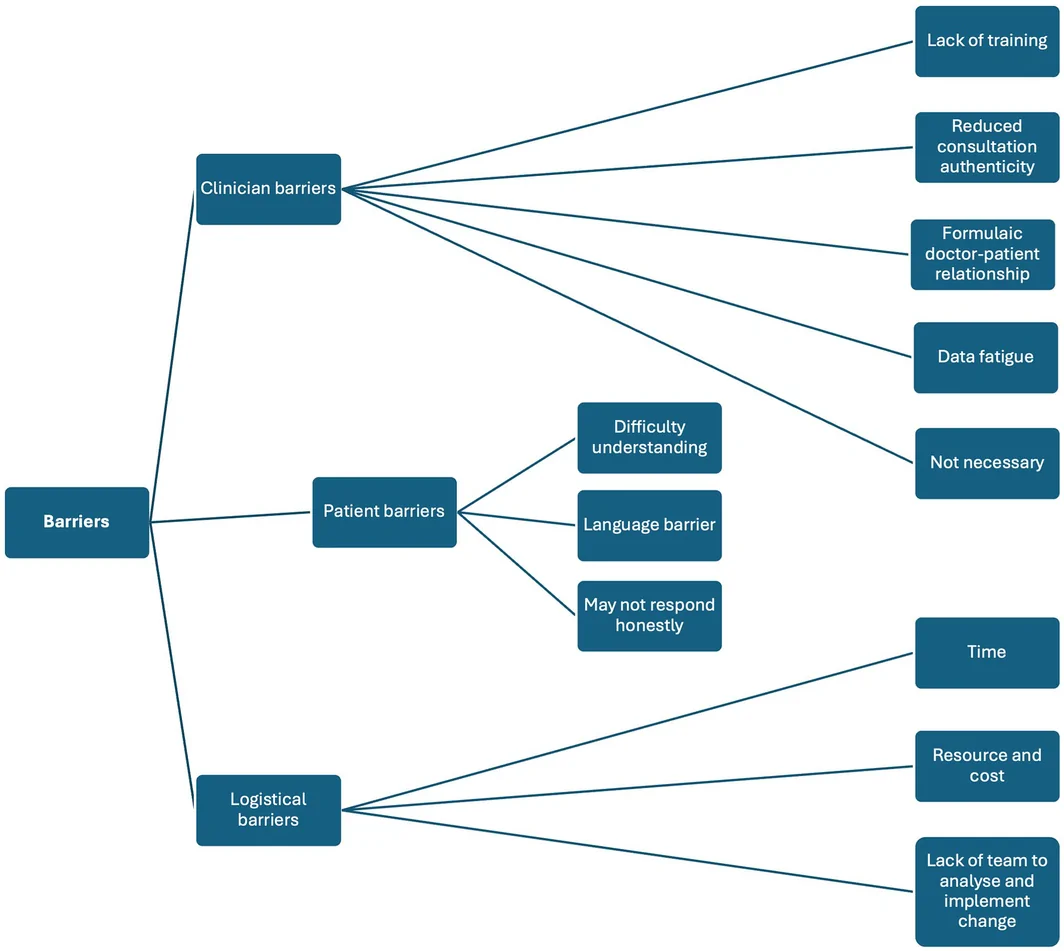
Reported barriers to utilization of formal quality of life (QoL) tools when evaluating quality of life in patients with head and neck cancer.

There were a variety of themes generated when participants were asked about any barriers to the use of formal QoL tools. These are summarized in Figure 2. Most participants commented on time, resources and cost being an issue, “you haven't got the time to sit and collect all the data” (P4‐OMFS), “time and cost” (P5‐OMFS), *“I think time is probably a big one, being able to sit down with them* (patients) *and fill out a proper questionnaire takes a lot of time*” (P6‐AAN). Other participants mentioned about losing the authenticity of a consultation if time is spent completing a questionnaire, “it does take something away from the kind of the…authenticity of the consultation” (P3‐ENT), “I don't know that we'd get a true picture from them either” (P6‐AAN). There were other comments mentioning barriers such as lack of time or resources to analyze and act upon the data, “who's going to analyze the results? Is there going to be any funding to institute change?” (P5‐OMFS), as well as a lack of training. In addition, some participants suggested formal tools may be difficult for patients to engage in in terms of understanding and language barriers, “I think sometimes the questionnaires can be a little bit wordy” (P8‐SLT), “there's language barriers, so they need to be translated for the patients…a lot of them can't read or write as well” (P6‐AAN).

## 4 Discussion

This paper used a qualitative, descriptive content analysis approach to explore perceptions of HCPs surrounding the importance and use of QoL instruments in the context of head and neck surgical oncology. Head and neck cancer patients are known to experience a disproportionate impact on their QoL across all domains (physical, social, functional and psychosocial) [3]. In addition, QoL outcomes have been linked to survival prediction among patients with head and neck cancer [17, 18]. Assessment of QoL in this patient group is therefore paramount to optimize not only patient experience, but also outcomes. It is therefore vital that HCPs involved in the head and neck oncology MDT are aware of the impact that QoL can have on their patients. While there is data exploring the relationship between patients and their own QoL, there is a sparsity of research surrounding the perception and use of QoL and formal tools from a multi‐disciplinary HCP standpoint in head and neck surgical oncology. Our findings highlight a notable disconnect between clinicians' recognition of QoL as an important outcome and the limited routine integration of formal QoL tools into multidisciplinary head and neck oncological practice.

Overall, there were several overarching categories that emerged from the 10 completed interviews. There was a general good understanding from HCPs of the meaning of QoL in a clinical context. There was an acknowledgement that QoL was related to a patient's ability to function across multiple domains, and that this was individual and unique to each patient. Most responses overlapped with the WHO definition of QoL as “an individual's perception of their position in life…in relation to their goals, expectations, standards and concerns” [19]. Generally, there was a feeling that the assessment of QoL is important, especially in the context of planning and offering treatment options and allowing patients to make informed decisions. This ties in with the existing evidence demonstrating the impact of QoL on head and neck cancer patients [20, 21, 22]. It is clear that clinicians acknowledge this impact and feel that it is significant enough to address in their consultations.

Participants also commented on the long‐term effects of radical surgery and how this can impact a patient long after a “cure” is achieved. This is especially crucial when offering surgical treatment options, with surgery shown to have significant effect on head and neck cancer patients' QoL [23]. In addition, most felt that the consideration of QoL was more important in oncology patients compared to non‐oncology patients. Again, this theme is largely reflected in the existing literature, with evidence suggesting that cancer patients have a different psychological burden [24] following treatment, including existential worries such as a feeling of loss of their sense of self and future [25]. Despite this, two participants felt that QoL was less important in their oncology practice compared to their non‐oncology work. This contrasted with the general feeling of the rest of the participants. Interestingly, both participants were ENT surgeons who had more than 15 years of experience as consultant head and neck surgeons. While no conclusions can be drawn regarding the influence of seniority itself, these responses may reflect differing clinical priorities or perspectives between survival and QoL from a clinician standpoint.

In terms of usefulness, most agreed that the use of formal QoL tools was valuable in clinical practice, for patients and in research contexts. This ties in with participant responses regarding their overall feeling that QoL is an important metric. Despite this, the overall evaluation of QoL during consultations was through informal measures. Therefore, although HCPs feel that evaluation of QoL is important, and formal QoL tools are useful, this does not translate to regular use of such QoL tools. Differences between professional groups were also apparent. The only participants routinely using formal QoL tools in practice were SLTs. In contrast, surgeons more commonly described informal conversational approaches to QoL assessment. This may reflect the differences between rehabilitation‐focused and surgically‐focused clinical workflows, consultation priorities, and perceived practicality of PROM integration between groups. Several explanations for this discrepancy emerged from the participant responses. Firstly, some clinicians felt that they were able to explore QoL adequately through use of informal measures, such as by simply conversing with patients and exploring their views, and therefore did not need to employ formal QoL instruments to gain more information. Secondly, and perhaps more importantly, this may occur due to various factors acting as perceived barriers to the use of formal QoL tools.

The most frequently mentioned barriers were “time” and “resources.” Many participants felt that the time taken to fill out QoL questionnaires was time that could be more valuably used talking to patients. A solution to this could be to ask patients to fill out these questionnaires prior to attending their appointments or in the waiting room. This is proposed in the existing literature where physicians have suggested that questionnaires could be nurse administered [26, 27]. When speaking to the two participants who frequently used formal QoL tools in their clinical practice, they mentioned that they often had patients complete QoL questionnaires in the waiting room prior to appointments. However, it was also mentioned that some patients experienced “questionnaire fatigue” that could limit the applicability of this approach. The use of questionnaires in clinical practice has been highlighted in previous research and reviews [28, 29]. Here, it was suggested that specific management guidelines were more likely to influence change than education of staff members. Our research adds to the growing body of evidence that can help demonstrate the perceived benefits of PROMs (patient reported outcome measures) among clinicians to policy makers to encourage change. Importantly, many barriers identified were structural rather than ideological. This distinction is important when designing future PROM implementation strategies.

Another potential solution to the aforementioned barriers is the use of electronic PROMs (ePROMs), which have increased in popularity in recent years [30], with computer‐based methods been shown to be robust, sustainable and acceptable to patients [31, 32, 33]. This can also minimize the workload from nurse‐led PROM collection, and automated scoring systems can flag concerns, changes or deteriorations prior to consultations without adding to clinician's cognitive load. Traditional paper‐based forms can be labor intensive, time consuming, and may be subject to bias or influence from clinicians. ePROMs have the potential to negate these effects and have been shown to have an excellent concordance with traditional PROMs when assessing xerostomia, dysphagia and QoL, even when used with older patients [34]. These approaches may address some of the barriers mentioned by participants, although their feasibility and implementation in this specific context would need to be evaluated and further considered.

The issue of resource in terms of “what would be done with any collected data” was also highlighted. Key themes that emerged included concerns regarding who would analyze the data, what would be the outcome of any analysis, and what changes would be implemented. These barriers could be addressed through the implementation of formal research teams who could satisfy these concerns. Often, clinical teams may not be involved in research, and so the utilization of formal and appropriately funded research teams can help bridge this gap to allow the transition of clinical data to be analyzed and acted upon appropriately.

To our knowledge, this is the first interview‐based multidisciplinary study evaluating clinician attitudes toward QoL specifically among the head and neck surgical oncology team at a single tertiary center. Previous studies related to this topic in head and neck cancer have either studied sustained use of a specific tool [33] or used surveys to gather opinions among head and neck surgeons [29, 35]. Importantly, such survey studies are subject to inherent sampling bias and other limitations including poor representation and recency bias [36]. Other research has also evaluated this outlook in different specialties. A recent study found clinicians caring for patients with HIV (Human Immunodeficiency Virus) felt assessing health‐related QoL could have a positive impact on patients in clinical and personal domains [26]. In addition, the findings from our study do agree with other existing literature on this subject where reviews have demonstrated a general positive attitude toward the use of PROMs in clinical practice [37, 38]. Furthermore, key barriers to the use of PROMs have been well documented. These are similar to the findings of our study and include aspects such as time constraints, patient compliance, unfitting infrastructure and a lack of clear pathway to action data [38, 39].

This study is not without its limitations. It can be argued that our sample size of 10 participants all from a single center limits the generalizability of our results. In addition, some members of the head and neck oncology MDT may be underrepresented. It is important to note that no member reflections/checking or decision logs were kept that may impact data trustworthiness. Finally, the nature of qualitative interviews lends itself to “social desirability bias” [40] where interviewees may be inclined to respond with answers in line with what is perceived to be the social norm, leading to misrepresentation of their true viewpoint. Despite these limitations, our sample size is informed by methodological guidance and aligned to qualitative studies with a similar methodology [9, 10, 11, 12] and was guided by information power with both authors agreeing that the dataset held sufficient depth and breadth to address the study aims. In addition, our study includes participants that are representative of all key members of the head and neck surgical oncology MDT, at a high‐volume tertiary referral center that encompasses a wide demographic in a large city of the United Kingdom. The emergent categories are therefore likely to be representative of the view of the head and neck surgical oncological MDT. Despite this, it is important to note that all participants from a single center may introduce institutional bias and limit perspective diversity.

There are various possible clinical applications of these findings. For example, there is a potential disconnect highlighted between clinician belief and clinical behavior. While HCPs largely agree that formal QoL is useful, they are not being routinely integrated into clinical practice. Despite most HCPs mentioning that they evaluate QoL through informal measures, they may be able to optimize a holistic, patient‐centered approach using both formal and informal methods. The lack of utilization of formal QoL instruments may contribute to an underassessment of patient distress and psychosocial needs. Formal QoL tools can facilitate structured communication and uncover potentially impactful functional, emotional, and social issues, especially in patients who struggle to articulate their concerns. Furthermore, the identified barriers in this work can help provide insight to the various clinician, patient, and logistical hurdles that limit the use of formal QoL instruments to allow further research to explore solutions to these barriers, some of which have been discussed. Nevertheless, their use is likely to be clinician dependent, and perhaps this work can spark conversation among head and neck surgical oncological MDTs to reflect more on this important topic and their assessment of their patients' QoL.

## 5 Conclusions

Overall, this study adds to the literature by providing an important analysis of perceptions of QoL and formal tools in head and neck surgical oncology from an underexplored multidisciplinary clinician perspective in this specific tertiary single‐center MDT. While there has been a research focus on QoL in recent years in this surgical group, this has largely been from a patient perspective. This article explores the thoughts, perceptions, and use surrounding QoL through a clinician lens. It highlights the clear disconnect between clinicians' recognition of QoL as an important outcome and the limited routine use of formal QoL instruments within the head and neck surgical oncology multidisciplinary practice. While clinicians across the MDT generally viewed QoL assessment positively, implementation of structured tools remained inconsistent and was influenced by structural barriers. These findings suggest that future efforts should focus on developing feasible, clinically embedded approaches to QoL integration within this context.

## Funding

The authors have nothing to report.

## Disclosure

The authors have nothing to report.

## Ethics Statement

This study was prospectively registered with Integrated Research Application System (IRAS) (343373) who confirmed its exemption from Research Ethics Committee (REC) approval.

## Conflicts of Interest

The authors declare no conflicts of interest.

## Supporting information


**Data S1:** Supporting Information A. Semi structured interview prompts.

## Data Availability

The data that support the findings of this study are available on request from the corresponding author. The data are not publicly available due to privacy or ethical restrictions.
