# Supplementary material for: Healthcare Practitioners' Perceptions of Quality‐of‐Life Tools in Head and Neck Surgical Oncology: A Qualitative Descriptive Content Analysis
Source: Laryngoscope Investig Otolaryngol. 2026 Aug 20;11(4):e70531. doi: 10.1002/lio2.70531 (PMC13492056; doi:10.1002/lio2.70531)
Supplement: Supplementary file 1 [file LIO2-11-e70531-s001.docx]

**Supplemental Material A**

**Semi-structured interview prompts**

The aim of these prompts are not to act as definitive questions that should be asked during the interview, rather a framework to help guide the interviewee through the process to help gain feedback surrounding their awareness, perceptions and utilisation of quality of life (QoL) and QoL tools during their own clinical practice specifically in the context of head and neck surgical oncology**.** Other questions can be asked depending on the responses of the interviewee. Questions should elicit content relevant to participants’ perceptions, awareness and use of QoL measures.

**Broad themes and main questions to help guide interviews:**

1. **Introduction**
   a. Remind participant that interview is voluntary and will be recorded for auto-transcription
   b. Invite honest reflection
   c. Ask if any questions prior to starting
2. Understanding of QoL
   a. What do you understand by QoL?
   b. Do you think it is important to consider patients QoL when planning and offering treatment options?
   c. Do you think QoL is more important in head and neck oncology than in other areas of your practice?
   d. Do you think your role places more or less QoL value than other members?
3. Awareness and utilisation of QoL
   a. Do you assess QoL when you see patients in clinic?
      i. Either formally or informally
   b. Are you aware of a) general and b) specific head and neck oncological QoL tools – if so which ones?
   c. Do you routinely use these in your clinical practice?
      i. If yes – which ones and why
      ii. If no – explore the barriers to their use
   d. Have they ever used them in other areas/centres?
4. Usefulness of QoL tools
   a. If yes – in what contexts?
   b. If no – why not?
      i. Explore if interviewee thoughts surrounding usefulness in a) clinical practice b) research c) for patients
5. Closing
   a. Anything else to add?

**Example probes**

1. Can you give me an example?
2. Can you tell me more about that?
3. What influenced that experience/thought?
